# Retraction Note: CDCA genes as prognostic and therapeutic targets in Colon adenocarcinoma

**DOI:** 10.1186/s41065-026-00756-w

**Published:** 2026-10-16

**Authors:** Zongquan Zhao, Xinwei Feng, Bo Chen, Yihong Wu, Xiaohong Wang, Zhenyuan Tang, Min Huang, Xiaohua Guo

**Affiliations:** 1 Department of General Practice, Pingjiang New Town Community Health Service, Center Sujin Street Gusu District, Suzho, 215000 Jiangsu China; 2 Department of Digestive Internal Medicine, Shanghai Changzheng Hospital, Shanghai, 200003 China; 3 Department of Oncology, Chengdu First People’s Hospital, Chengdu, 610041 Sichuan China; 4 Department of General Practice, Runda Community Health Service Center, Wumenqiao Street, Gusu District, Suzhou, 215000 Jiangsu China; 5 Department of General Practice, Community Health Management Center of Suzhou Municipal Hospital, Suzhou, 215000 Jiangsu China; 6 Department of General Practice, Suzhou Municipal Hospital, Suzhou, 215000 Jiangsu China; 7 Department of Digestive Surgery, Xi’an Jiaotong University School of Medicine Affiliated Honghui Hospital, Xi’an, 700054 Shaanxi China

**Retraction Note: Hereditas 162, 19 (2025)**

**https://doi.org/10.1186/s41065-025-00368-w**

The Editor-in-Chief has retracted this article. After publication, concerns were raised regarding the in vitro images presented in Figs. 10 and 11 of this article, where the patterns of cells and colonies do not appear consistent with natural cell growth and proliferation. Additionally, the uncut western blot gel presented in the Supplementary Material appears to show contradictory results, with two proteins probed in adjacent lanes with different results. It has also been noted that a number of references in this article do not appear to support the statements preceding the citations.

The Editor-in-Chief therefore no longer has confidence in the presented data.

The authors have not responded to correspondence regarding this retraction.

